# Chemoimmunotherapy in advanced esophageal squamous cell carcinoma: optimizing chemotherapy regimens for immunotherapy combinations

**DOI:** 10.1038/s41392-022-01077-w

**Published:** 2022-07-13

**Authors:** Zhichao Liu, Jun Liu, Zhigang Li

**Affiliations:** 1 Department of Thoracic Surgery, Shanghai Chest Hospital, Shanghai Jiao Tong University School of Medicine, Shanghai, China; 2 Esophageal Diseases Center, Shanghai Jiao Tong University, Shanghai, China; 3 Department of Radiation Oncology, Shanghai Chest Hospital, Shanghai Jiao Tong University School of Medicine, Shanghai, China

**Keywords:** Gastrointestinal cancer, Cancer therapy

A recent study published in BMJ by Lu et al.^1^ has reported the interim findings from the ORIENT-15, which is a global, multi-center and randomized phase 3 trial to compare chemoimmunotherapy to chemotherapy alone as first-line treatment in advanced esophageal squamous cell carcinoma (ESCC), and provoked thinking about the chemotherapy regimens for chemoimmunotherapy combinations.

Immune-checkpoint blockades (ICB) have been added to chemotherapy in advanced esophageal cancers in multiple clinical trials. The KEYNOTE-590 (pembrolizumab), ESCORT-1st (camrelizumab), CheckMate-648 (nivolumab), JUPITER-6 (toripalimab), and ORIENT-15 (sintilimab)^1^ trials all showed superior response and survival for chemoimmunotherapy. These results provided solid evidence for chemoimmunotherapy as first-line treatment of advanced ESCC. Since the current strategy is merely adding ICB to standard-of-care chemotherapy regimens, the next focus should be optimizing chemoimmunotherapy regimens based on synergistic mechanism to further improve clinical efficacy.

**Fig. 1:**
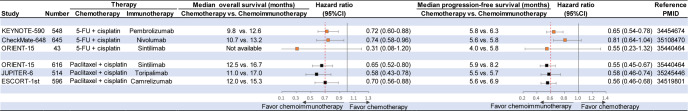
Forest plot analysis of overall survival and progression-free survival in advanced esophageal squamous cell carcinoma for the latest phase 3, first-line trials comparing immunotherapy or placebo in combination with chemotherapy

For advanced ESCC, first-line chemotherapy regimens include paclitaxel plus cisplatin (PC), which is a common backbone chemotherapy (BBC) used in China, and 5-fluorouracil plus cisplatin (CF), which is a common BBC used in Western countries and Japan. The efficacy and median overall survival (OS) appear generally comparable across the regimens in ESCC.^2^ Of note, BBC regimens differed in each of the above-mentioned published trials in advanced ESCC. The KEYNOTE-590 and CheckMate-648 trials adopted CF regimen, while the ESCORT-1st, JUPITER-6 and ORIENT-15 mainly adopted PC regimen. If we consider the BBC regimens for ICB, are there any differences among these trials (Fig. 1)? For CF-based BBC studies (KEYNOTE-590 and CheckMate-648 have similar dose intensity of CF chemotherapy), the median OS of chemotherapy group was about 10 months (9.8–10.7), and that of chemoimmunotherapy was about 13 months. The hazard ratio (HR) for chemoimmunotherapy relative to chemotherapy alone is around 0.72–0.74. As for PC-based BBC studies (ORIENT-15, JUPITER-6, and ESCORT-1st have similar dose intensity of PC-chemotherapy), the median OS of chemotherapy group ranged from 11 to 12.5 months; and that of chemoimmunotherapy group was increased at least to 15 months, with a more than 30% reduction risk of overall death (HR: 0.58–0.70).

Because of the differences in study design, a reasonable comparison of these studies is difficult; but we can obtain some important information. The median OS in chemotherapy group were all around 10–12 months regardless of CF or PC regimen. However, in chemoimmunotherapy group, ICB + PC appeared to have a better reduction in overall mortality risk (HR ≤ 0.70, median OS ≥ 15 months). Similarly, for progression-free survival, ICB + PC seemed to have relatively more combination benefit compared with ICB + CF. For the current studies, only ORIENT-15 study^1^ was designed with stratification of chemotherapy regimens and results showed that HR for OS of ICB + PC vs. PC and ICB + CF vs. CF were 0.65 and 0.31, respectively. Of note, the HR of ICB + CF subgroup was not statistically significant, which may be due to the small number of patients (7% of total). However, this subgroup analysis from ORIENT-15 and the above observation lead to an interesting speculation that different BBC regimens combined with ICB may have different effects. Because population characteristics differ between these trials, further investigations comparing chemoimmunotherapy-combination regimens are needed. RATIONALE-306 trial is being conducted globally with different BBC regimens, and the results will provide essential clinical data for future work. Moreover, a retrospective comparison of CF/PC BBC plus ICB using propensity-score matched cohorts based on real-world data may be a feasible alternative to provide important reference.

Growing evidence suggest that the efficacy of ICB treatment is related to tumor microenvironment (TME). Indeed, the effects of chemotherapeutic drugs on TME and immune cells are not fully understood, which may affect immunotherapy efficacy. So far we have no direct evidence to support this speculation in ESCC. However, recent studies on chemotherapy modulating the immune TME have observations related to this speculation. The study by Zhang et al. performed single-cell sequencing from patients with advanced triple-negative breast cancer treated with paclitaxel chemotherapy alone or in combination with ICB.^3^ This study proposes an antagonistic effect of paclitaxel on ICB based on the immunosuppressive changes in immune cell following paclitaxel treatment. This observation may account for the recent clinical data that no significant benefit from chemoimmunotherapy combination compared to paclitaxel alone.^4^ In a recent study of ESCC, single-cell sequencing was applied to investigate the effects of PC-chemotherapy on TME.^5^ The immune cell dynamics showed cellular and molecular shifts towards immune-activation TME following PC-chemotherapy. CD8+ T-cell levels was significantly increased and the proportion of CD8+PD1+ T cells was elevated, which is important feature of predicting response to anti PD-1/PD-L1 immunotherapy. Larger proportions of plasma cells and monocytes were also observed, which have been demonstrated to be associated with response to ICB. Furthermore, macrophage following PC-chemotherapy highly expressed genes involved in pathways of anti-tumor (e.g., interferon-gamma and interferon-alpha) response, indicating a M1-like polarization phenotype. This result provides a theoretical basis for that ICB + PC combination can have synergistic anti-tumor effect. This might be a potential explanation for the observation that ICB + PC exhibited relatively superior efficacy in the first-line treatment of ESCC. Indeed, previous studies also demonstrated a different trend in pathological response in ESCC patients receiving PC or CF plus radiotherapy, suggesting a different impact of chemotherapeutic regimes on TME. Currently, we have no evidence of effect of CF chemotherapy on immune microenvironment in ESCC, therefore, it is difficult to say whether there is a synergistic difference between PC and CF plus ICB.

Chemoimmunotherapy has arrived in clinical setting and will stay for years to come, however, chemotherapy regimens have not been optimized for such combination in ESCC. Improving the efficacy of chemoimmunotherapy will hinge on better understanding the synergistic and antagonistic effects of chemotherapy on immunotherapy. The next step is to focus on dissecting mechanisms as well as to optimizing/individualizing treatments. There are many possible research directions worthy of exploring, including the impact of BBC regimes on TME, the sequence of chemotherapy and ICB on efficacy, the effect of dose intensity of chemotherapy on immunity, and predictive biomarkers. Future studies are warranted to collect more detailed data on these important issues.
